# Disruption of Vascular Barriers in Tumors and in the Brain

**DOI:** 10.1186/2050-5736-3-S1-O15

**Published:** 2015-06-30

**Authors:** Nathan McDannold

**Affiliations:** 1 Brigham & Women’s Hospital/Harvard Medical School, Boston, MA, United States

## Background/introduction

While most brain tumors do not have an intact blood-brain barrier (BBB), vascular barriers in the tumor and the surrounding intact brain tissue are major challenges for the use of chemotherapy for CNS malignancies. The permeability of brain tumor vessels can be highly heterogeneous, and factors such as high interstitial pressures can prevent agents from reaching an effective concentration in all tumor regions. Furthermore, tumors such as glioma are highly infiltrative, and these infiltrating tumor cells in the brain tissue surrounding the vascular portion of the tumor are protected by the BBB.

## Methods

Numerous studies have demonstrated that when combined with an ultrasound contrast agent, ultrasound can temporarily disrupt the BBB and increase the delivery of agents across the “blood-tumor barrier”. This presentation will review data obtained in rat and mice brain tumor models that evaluated tumor growth rates and survival after ultrasound-enhanced chemotherapy delivery. These studies have evaluated a range of primary and metastatic models and drugs.

## Results and conclusions

These studies have all demonstrated that ultrasound-induced permeabilization of the BTB and chemotherapy can improve outcomes in rodent and human tumor models. Drugs including BCNU, doxorubicin (both free and liposomal), temozolimide, and trastuzumab have been tested. While in some cases the response has been modest, with multiple treatments dramatic improvements have been reported.

These studies demonstrate the potential for this technology, either alone or in combination with focused ultrasound ablation, to provide more effective treatment options for patients with brain tumors. The next steps needed for clinical translation, including the need for tests in better tumor models, improvements to transcranial focused ultrasound systems to enable sonication of large volumes, and the development of effective methods to monitor and guide the procedure outside the MRI will be discussed.

